# Commercial Price Variation for Common Cardiovascular Services Across 4 Major US Insurers

**DOI:** 10.1001/jamanetworkopen.2026.23326

**Published:** 2026-07-16

**Authors:** Alexander P. Philips, Sanket S. Dhruva, Christopher Whaley

**Affiliations:** 1 Center for Advancing Health Policy Through Research, Brown University School of Public Health, Providence, Rhode Island; 2 Warren Alpert Medical School of Brown University, Providence, Rhode Island; 3 Department of Health Services, Policy, and Practice, Brown University School of Public Health, Providence, Rhode Island; 4 Section of Cardiology, Department of Medicine, University of California, San Francisco, San Francisco

## Abstract

**Importance:**

Cardiology services are among the most frequently used and costly services and procedures in US health care. Understanding price variation is essential for employers, patients, and policymakers to make informed decisions, contain costs, and promote value-based care.

**Objective:**

To assess the extent of variation in commercial insurance payment rates for common cardiology services across major US insurers and states using newly available Transparency in Coverage (TIC) data.

**Design, Setting, and Participants:**

This cross-sectional study used April 2025 TIC data from a third-party vendor reflecting 2023 contract year claims. The dataset included approximately 6.7 million professional and 104 563 facility price points for 32 common cardiology services across 4 major commercial insurers (Blue Cross Blue Shield, UnitedHealthcare, Aetna, and Cigna Healthcare), representing 78% of the commercial insurance market. The sample covered 51 568 physicians and 4128 facilities nationwide.

**Exposure:**

Negotiated allowed amounts paid by 4 major insurers for cardiology services, analyzed by *Current Procedural Terminology* code and grouped into categories such as diagnostic imaging, electrophysiology, and stress testing.

**Main Outcomes and Measures:**

Variation in allowed payment amounts for each cardiology service across insurers and states. Measures included median prices and IQRs, coefficients of variation, and volume-weighted price indices.

**Results:**

Facility negotiated price variation exceeded professional fee variation, with a median interquartile ratio of 2.56 across services. For example, median facility fees for implantable cardioverter-defibrillator (ICD) insertion ranged from $6674 (IQR, $3069-$11 402) to $36 269 (IQR, $18 673-$66 880) across insurers. Geographic variation was also substantial, with a median facility price for coronary angiography of $7683 (IQR, $4561-$10 101) across states. Blue Cross Blue Shield had the highest mean facility prices while Aetna had the lowest across most service categories.

**Conclusions and Relevance:**

In this cross-sectional study of commercial insurance payments for cardiology services, there was substantial variation across both insurers and states, particularly in facility fees. These findings reveal the influence of payer contracting strategies and market dynamics and underscore the need for greater transparency and regulatory oversight to promote efficient health care spending.

## Key Points

**Question:**

How do commercial insurance payment rates for common cardiology services vary across insurers and states in the US?

**Findings:**

In this cross-sectional study of April 2025 Transparency in Coverage price data collected by a third-party vendor from 4 major insurers, facility prices for cardiology services showed substantially greater variation than professional fees. Median facility prices for some procedures—such as implantable cardioverter-defibrillator insertion—ranged from $6674 to $36 269 across payers, with a median facility price for coronary angiography of $7683 across states.

**Meaning:**

These findings underscore the need for greater oversight and policy regulations to enhance price transparency and curb excessive spending.

## Teaser

This cross-sectional study examines Transparency in Coverage price data for common cardiology procedures from 4 large national insurers.

## Introduction

Commercial insurers provide health insurance coverage for approximately half of all people in the US. Commercial rates are established through individual negotiations that lack transparency, resulting in variable payment rates.^1,2^ This process has precluded regulators from monitoring prices and employers and other purchasers from negotiating better rates or steering patients toward more cost-effective care.

In 2023, implementation of Hospital Price Transparency and Transparency in Coverage (TIC) rules mandated insurers to publicly disclose negotiated rates for health care services. These policies aim to improve market functioning by equipping stakeholders with data to compare prices across clinicians, facilities, and payers. Given clinician concerns with insurer strategies on reimbursement, prior authorization, and the care of patients with multiple sources of insurance and varying negotiated rates, it is important for clinicians to have data on and understand variations in payment across insurers. Additionally, both purchasers and policymakers use price information to design initiatives that ensure sustainable health care costs.^3,4^

Cardiology services are among the most frequently utilized and costly in US health care. In 2021 alone, cardiovascular conditions led to nearly 4.7 million hospital admissions nationwide, generating an estimated $108 billion in total expenses (adjusted to 2023 dollars).^5^ Among these, hospitalizations for heart failure incurred the greatest financial burden at $18.5 billion, while admissions for non–ST-elevation myocardial infarction followed at $11.2 billion, and stroke followed at $10.9 billion.^5^ Previous literature has documented price variation in cardiology using hospital chargemaster data^6,7^ or for specific services and hospitals^8^; however, price variation for common cardiology services has not been examined globally using data reported by large commercial payers. Accordingly, we used TIC data to examine variation in commercial insurance payment rates for common cardiology services.

## Methods

The Brown University institutional review board deemed this study exempt from ethics review and informed consent since no patient data were included. Reporting of results adhered to Strengthening the Reporting of Observational Studies in Epidemiology (STROBE) guidelines for cross-sectional studies.

In this cross-sectional study, we examined April 2025 TIC price data collected by a third-party vendor, ClarifyHealth, for common cardiology services from 4 large national insurers: Blue Cross Blue Shield (BCBS), UnitedHealthcare, Cigna Healthcare (Cigna), and Aetna. Collectively these insurers constitute 78% of the commercial insurance market share.^9^ We analyzed commercial prices (excluding Medicare Advantage) for physicians with billed claims during the 2023 contract year for 32 common cardiology services. We selected these services based on their frequency in commercial claims data. We broadly classified them as stress testing, diagnostic imaging, cardiac electrophysiology, or interventional cardiology. Prices reflect the allowed amount that is negotiated between an insurer and physician for a given *Current Procedural Terminology* code (distinct from the cash price and the chargemaster rate)^10^ and includes both payments from the insurer and patient cost-sharing responsibilities. In sum, the unit of observation is the total price for a unique payer-provider–*Current Procedural Terminology* code combination. Data are weighed by provider claim volume to address variation in provider market share.

For professional fees, our sample included approximately 6.7 million unique price points for 51 568 physicians (primarily cardiologists and radiologists for some cardiac imaging services). For facility fees, our sample included 104 563 unique price points and 4128 facilities.

### Statistical Analysis

We analyzed distributional differences in prices (mean, median, and percentiles), coefficients of variation, and volume-weighted price indices by payer. The price index is the weighted mean ratio of each procedure’s insurer-specific price to the mean price for each procedure across all insurers. This price index measures the deviations from the national average price, after accounting for differences in procedure spending. An index of 1.00 represents that the insurer’s price is at the average, while an index below 1.00 represents lower-priced procedures, and an index above 1.00 represents higher-priced procedures (explained further in the eMethods in Supplement 1). We also examined geographic variation by state for median prices of invasive coronary angiography. Data analysis was performed using R, version 4.4.1 (R Program for Statistical Computing).

## Results

Facility pricing for cardiology procedures exhibited more variation than professional pricing across 25 of 26 services with a facility charge (eTable 1 and eTable 2 in Supplement 1). These differences were most pronounced for diagnostic imaging and stress testing. For example, transthoracic echocardiogram facility fees had a coefficient of variation (SD divided by mean) of 3.87 compared with the corresponding professional fee variation of 0.61.

There was substantial facility price variation in cardiology procedures, with a median interquartile ratio of 2.56 across all payers. The highest facility interquartile ratio was for UnitedHealthcare’s ICD placement (3.58). Median facility fees for permanent pacemaker insertion ranged from $6366 (IQR, $2775-$10 714) for Aetna to $17 678 (IQR, $11 435-$30 304) for UnitedHealthcare, while median facility fees for ICD placement ranged from $6674 (IQR, $3069-$11 402) for Aetna to $36 269 (IQR, $18 673-$66 880) for UnitedHealthcare.

Price variation in professional fees was also notable but comparatively smaller, with a median interquartile ratio for a given payer and service of 1.78 across all payers. The highest professional interquartile ratio was for Cigna’s myocardial positron emission tomographic stress test (3.02; median professional fee, $444 [95% CI, $253-$764]). Across payers, for permanent pacemaker insertion, median professional fees ranged from $344 (IQR, $261-$496) for Aetna to $885 (IQR, $665-$1179) for UnitedHealthcare, while for ICD placement, median professional fees spanned from $610 (IQR, $460-$935) for Aetna to $1581 (IQR, $1182-$2111) for UnitedHealthcare.

**Figure 1.**
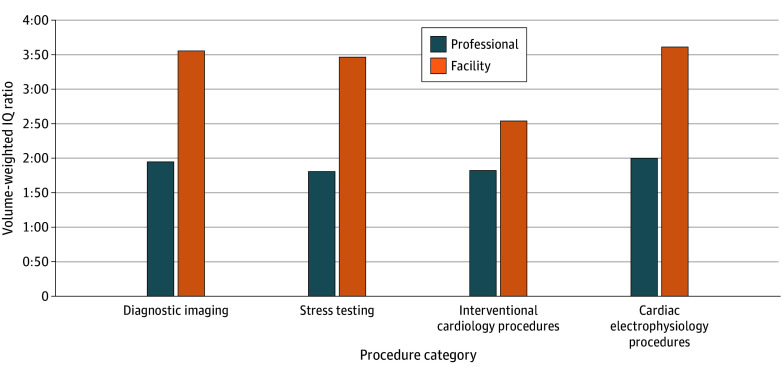
Bar Graph of Volume-Weighted Interquartile (IQ) Ratios of Price Across 4 Major Cardiology Procedure Categories

As volume-weighted price indices by payer (Table) are interpretable as a percentage deviation from the 4-insurer average, the observed facility indices suggest economically meaningful variation, ranging from 0.39 (61% below the market average) for Aetna professional prices for cardiac electrophysiology procedures to 1.46 (46% above the market average) for UnitedHealthcare facility prices for stress testing. Price indices showed that BCBS consistently had the highest facility prices for cardiology services, with indices across service categories as much as 1.31 times the market average. In contrast, Aetna’s facility prices were the lowest, as little as 0.39 times the market average for cardiac electrophysiology procedures. UnitedHealthcare and Cigna displayed mixed patterns, with UnitedHealthcare’s facility fees for stress testing peaking at 1.46, and Cigna’s interventional cardiology facility fees at 1.30. The greatest variation was seen in facility fees for cardiac electrophysiology procedures, where BCBS charged over 3 times more than Aetna. Volume-weighted interquartile ratios of price across 4 major cardiology procedure categories are presented in Figure 1.

**Table.** Professional and Facility Price Indices by Payer for Cardiology Services

Payer source	Price index^a^										
	Professional						Facility				
	Cardiac electrophysiology		Diagnostic imaging	Interventional cardiology procedures	Stress testing	Overall	Cardiac electrophysiology procedures	Diagnostic imaging	Interventional cardiology procedures	Stress testing	Overall
	Evaluation	Procedures									
All 4 payers^b^	1.00	1.00	1.00	1.00	1.00	1.00	1.00	1.00	1.00	1.00	1.00
Aetna	0.60	0.39	0.78	0.77	0.77	0.72	0.39	0.43	0.78	0.44	0.51
BCBS	1.09	0.98	1.06	1.01	1.07	1.04	1.31	1.31	1.04	1.27	1.23
Cigna Healthcare	1.04	0.89	0.96	1.01	0.92	0.96	1.07	0.83	1.30	1.20	1.11
UnitedHealthcare	1.03	1.29	0.99	1.06	1.02	1.05	1.14	0.83	1.09	1.46	1.09

Abbreviation: BCBS, Blue Cross Blue Shield.

^a^ Indicates volume-weighted ratios of each insurer’s negotiated prices to the mean price across all 4 insurers for the same procedures.

^b^ Set to 1.00; values below 1.00 indicate prices below the 4-insurer average, and values above 1.00 indicate prices above the average. For example, an index of 1.31 indicates prices 31% higher than average, while an index of 0.39 indicates prices 61% lower than average. The methodology for calculating these volume-weighted price indices is shown in the eMethods in the Supplement.

**Figure 2.**
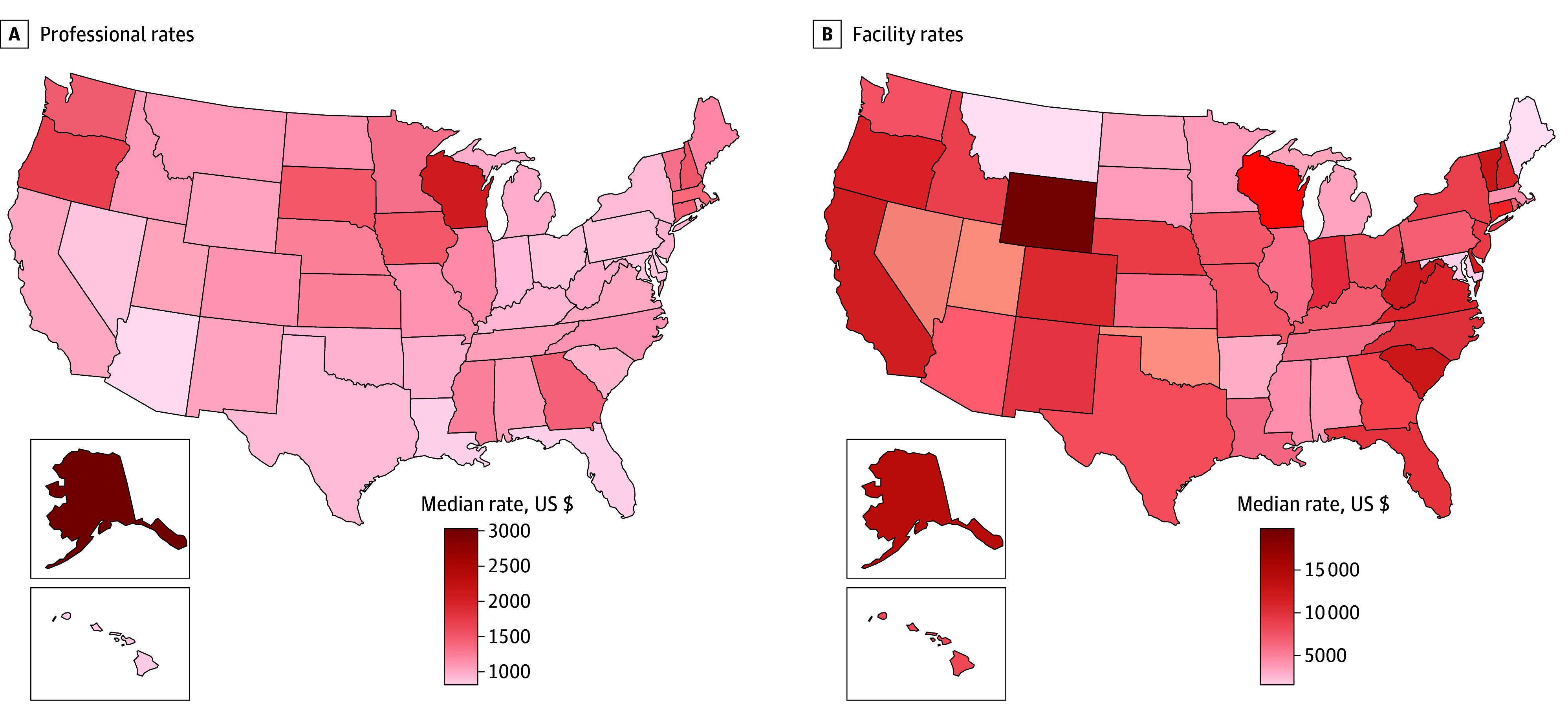
Map of Median Rates by State for Invasive Coronary Angiogram With Left Heart Catheterization *Current Procedural Terminology* code is 93458. Findings are based on Transparency in Coverage data from 4 major insurers representing 78% of the commercial market; all states have substantial commercial enrollment.

Figure 2 maps the variation in prices for invasive coronary angiography with left heart catheterization across states as a descriptive example of price variation for a cardiac procedure by geography. Median professional price was $1045 (IRQ, $932-$1261) across states, and median facility price was $7683 (IQR, $4561-$10 101) across states.

## Discussion

We found substantial variation in commercial pricing for common cardiology procedures, with greater variability in facility than professional prices. These findings build on prior analyses by capturing not only geographic but also payer-specific differences, underscoring the influence of unique negotiation strategies among insurers. There are multiple hypothesized mechanisms for these differences between insurers. BCBS had the highest facility price indices across electrophysiology and diagnostic imaging, possibly reflecting a commitment to broad provider networks, which often necessitate higher facility reimbursements to ensure comprehensive access.^11,12^ Cigna, in contrast, maintained lower facility prices for diagnostic imaging and interventional cardiology, suggesting effective selective contracting with facilities willing to accept lower rates in exchange for increased patient volume.^11^ Aetna’s pattern of the lowest professional and facility price indices across nearly all cardiology services may point to an aggressive negotiation approach, potentially prioritizing cost containment over network breadth.^11^ UnitedHealthcare exhibited a distinctive profile, with a high professional price index for cardiac electrophysiology procedures and the highest facility price index for stress testing, which may reflect a targeted strategy to secure specialized provider relationships and leverage vertical integration to manage facility costs.^13^

Our findings of variation in facility fees for cardiology procedures compared with professional fees may reflect, in part, differences in site-of-service utilization. Some of these procedures can often be performed across multiple settings (hospitals, ambulatory surgical centers, and physician offices). While our current analysis focuses on payer-specific variation rather than site-of-service differences, the observed price patterns—particularly the wider variation in facility fees—may reflect these site-of-service dynamics. Future research should examine how imaging prices vary across different care settings and how site-neutral payment policies might affect commercial market prices.

Price variation in cardiology is particularly relevant due to the increasing prevalence of cardiac conditions in an aging population and novel technologies being introduced that are high cost. The geographic variation in prices further underscores the influence of local market dynamics, such as hospital consolidation, competition, and regional cost structures, on health care pricing. Outlier states (such as Wisconsin, Alaska, and Wyoming) with higher-than-average prices may be explained by physician practices with high market share^2^ or Medicare payment policy for rural areas.^14^

### Limitations

This study has some limitations. Cross-sectional data may not have captured seasonal variations, longitudinal trends, or recent market changes. We also could not track how much of the difference in payments translates to differences in physician compensation. Our findings of greater variation in facility fees may also reflect differences in site-of-service utilization, as cardiology services can sometimes be performed across multiple settings; we cannot stratify our price analysis by site-of-service principally due to differential reporting across payers. Additionally, incentives created by payer reimbursement policies have driven migration of services from lower-cost office settings to higher-cost hospital outpatient departments, further amplifying facility price variation.^15^

 If price variation reflects clinical or perceived quality variation, purchasers and policymakers must find the balance between receiving higher-quality care and spending financial resources elsewhere. However, existing studies show no relationship between prices and quality or efficiency of care.^7,16^ If price variation is instead driven by consolidation or anticompetitive contracting, then regulators should design policies that ensure competitive health care markets. The factors determining price variation are likely somewhere in the middle of these 2 possibilities.

## Conclusions

In this cross-sectional study of commercial insurance payments for cardiology services, there was substantial variation across both insurers and states, particularly in facility fees. Future work should examine the underlying causes of price variation in cardiology.

## Notes

Supplement 1. **eMethods.** Price Index Construction **eTable 1.** Mean Prices and Price Variation for 32 Common Cardiology Services **eTable 2.** Mean Prices and Price Variation for 32 Common Cardiology Services by Payer

Supplement 2.
Data Sharing Statement
