# Supplementary material for: Commercial Price Variation for Common Cardiovascular Services Across 4 Major US Insurers
Source: JAMA Netw Open. 2026 Jul 16;9(7):e2623326. doi: 10.1001/jamanetworkopen.2026.23326 (PMC13377394; doi:10.1001/jamanetworkopen.2026.23326)
Supplement: Supplement 1. — eMethods. Price Index Construction eTable 1. Mean Prices and Price Variation for 32 Common Cardiology Services eTable 2. Mean Prices and Price Variation for 32 Common Cardiology Services by Payer [file jamanetwopen-e2623326-s001.pdf]

Supplementary Online Content

Phillips AP, Dhruva SS, Whaley C. Commercial price variation for common cardiovascular services across 4 major US insurers. *JAMA Netw Open.* 2026;9(7):e2623326. doi:10.1001/jamanetworkopen.2026.23326

eMethods. Price Index Construction
eTable 1. Mean Prices and Price Variation for 32 Common Cardiology Services
eTable 2. Mean Prices and Price Variation for 32 Common Cardiology Services by Payer

This supplementary material has been provided by the authors to give readers additional information about their work.

© 2026 Phillips AP et al. *JAMA Network Open*.

eMethods. Price Index Construction

The Transparency-in-Coverage (TiC) data contain detailed information on insurer- and provider-specific negotiated rates. Discount-off-charges contracts are handled by ClarifyHealth using adjudicated claims to infer CPT-level allowed amounts. Contracts for which a valid CPT-level rate cannot be established are excluded. Modifier-level rate variation is retained when reported in TiC files, and volume-weighting mitigates reliability concerns from modifier-related rate variation.

To make these rates interpretable and single-dimensional, we construct a price index that aggregates the full set of weights into a single metric. This single metric allows for a more straightforward comparison of rates across insurers and geographies.

To construct this price index metric, we use existing approaches that have constructed similar price measures using medical claims data (e.g. Dunn, Shapiro, and Liebman 2013; Neprash et al. 2015). We define procedure-specific weights (w) for CPT code c as:

$$w_c = \frac{price_c * \ q_c}{\sum_{c\,=\,1}^{C\,=\,32} \quad (price_c * \ q_c)}$$

These weights represent the aggregate share of spending accounted for each procedure code (c). Intuitively, each procedure's weight represents the spending on that code as the annual share of spending across the total thirty-two selected procedures. To construct total spending (price times quantity (q)), for both each individual procedure code and across all 32 codes, we use the commercial claim volume data from Clarify.

Next, for each insurer b, we calculate the price index as:

$$index_b = \sum_{b\,=\,1}^{B\,=\,4} \frac{price_{cb}}{price_c} * \ w_c$$

This index is the weighted average ratio of each procedure's insurer-specific price ($price_{cb}$) to the mean price for each procedure across all insurers $price_c$. The weights (w_c) calculated earlier adjust for differences in total spending (e.g., procedures that account for a larger portion of total spending are adjusted upwards). Intuitively, this price index measures the deviations from the national average price, after accounting for differences in procedure spending. An index of 1.0 represents that the insurer's price is at the average, while an index below 1.0 represents lower-priced procedures, and above 1.0 represents higher-priced procedures.

eTable 1. Mean Prices and Price Variation for 32 Common Cardiology Services

				Professional Prices				Facility Prices			
**Procedure Category**	**Description**	**Service Code**	**Unique Providers**	**Mean (SD)**	**Median (interquartile ratio)**	**Ratio of 75th to 25th %tiles**	**Coefficient of Variation**	**Mean (SD)**	**Median (interquartile ratio)**	**Ratio of 75th to 25th %tiles**	**Coefficient of Variation**
Cardiac Electrophysiology Evaluation	Electrocardiogram	93010	29944	16 (11)	13 (10 - 19)	1.98	0.66				
	30-day ECG* monitor	93228	11236	46 (27)	39 (30 - 53)	1.77	0.58				
	Dual chamber pacemaker programming	93280	10355	82 (45)	69 (52 - 94)	1.81	0.55				
	Single chamber ICD* programming	93282	6313	87 (46)	74 (56 - 101)	1.81	0.53				
	Remote interrogation - pacemaker	93294	8726	55 (30)	47 (36 - 62)	1.76	0.54				
	Remote interrogation - ICD*	93295	7702	84 (56)	69 (52 - 97)	1.85	0.67				
Cardiac Electrophysiology Procedures	Dual-chamber permanent pacemaker insertion	33208	4967	954 (662)	801 (601 - 1104)	1.84	0.69	17178 (15573)	13035 (6920 - 22132)	3.2	0.91
	ICD* placement	33249	3670	1626 (1378)	1359 (1000 - 1915)	1.91	0.85	34456 (36080)	22544 (8150 - 49877)	6.12	1.05
	ILR* placement	33285	5625	5819 (5068)	4348 (2853 - 7374)	2.58	0.87	11487 (10038)	9291 (4685 - 15081)	3.22	0.87
	LAAO*	33340	2203	1389 (766)	1201 (903 - 1666)	1.84	0.55	8072 (8113)	5922 (3021 - 9751)	3.23	1.01
	Cardioversion	92960	14272	283 (189)	241 (171 - 333)	1.95	0.67	2369 (4556)	1650 (816 - 2698)	3.3	1.92
	Supraventricular tachycardia ablation	93653	3059	1630 (1053)	1374 (1000 - 1929)	1.93	0.65	23228 (42762)	16191 (8497 - 30234)	3.56	1.84
	VT* ablation	93654	1838	2117 (1240)	1824 (1311 - 2579)	1.97	0.59	24152 (52864)	16274 (8529 - 31216)	3.66	2.19
	Atrial fibrillation ablation	93656	3106	2101 (1348)	1780 (1298 - 2526)	1.95	0.64	23462 (42946)	16372 (8800 - 30890)	3.51	1.83

© 2026 Phillips AP et al. *JAMA Network Open*.

Diagnostic Imaging	Cardiac MRI*	75561	3869	686 (550)	526 (362 - 810)	2.24	0.8	1614 (4335)	1037 (691 - 1719)	2.49	2.69
	Coronary calcium scan	75571	7971	135 (121)	100 (71 - 157)	2.2	0.9	863 (4834)	254 (149 - 572)	3.85	5.6
	CCTA*	75574	9042	503 (408)	388 (273 - 568)	2.08	0.81	1153 (3302)	686 (456 - 1120)	2.46	2.86
	Transthoracic echocardiogram	93306	30767	287 (175)	231 (173 - 341)	1.97	0.61	1325 (5126)	643 (318 - 1229)	3.87	3.87
	Transesophageal echocardiogram	93312	14183	322 (194)	272 (206 - 374)	1.82	0.6	1251 (4900)	642 (365 - 1193)	3.27	3.92
	Carotid ultrasound	93880	26378	227 (163)	180 (137 - 253)	1.85	0.72	830 (4409)	419 (222 - 692)	3.12	5.31
Interventional Cardiology Procedures	PAP* sensor placement	33289	517	621 (333)	524 (417 - 728)	1.75	0.54	24973 (32560)	14993 (8109 - 33232)	4.1	1.3
	TAVR*	33361	2098	2177 (1244)	1886 (1336 - 2642)	1.98	0.57	7454 (6672)	6018 (3467 - 8975)	2.59	0.9
	TMVR*	33418	794	3379 (1913)	2817 (1996 - 4168)	2.09	0.57	6191 (7348)	4704 (2606 - 6524)	2.5	1.19
	Intra-aortic balloon pump placement	33967	3497	501 (575)	395 (295 - 547)	1.86	1.15	13785 (14984)	8086 (3963 - 18098)	4.57	1.09
	Percutaneous LVAD* placement to left heart	33990	2614	665 (368)	576 (425 - 803)	1.89	0.55	6754 (7267)	4799 (2397 - 8630)	3.6	1.08
	PCI* with stent placement	92928	9079	1034 (718)	882 (655 -1194)	1.82	0.69	16080 (12945)	12710 (7266 - 20385)	2.81	0.81
	Right heart catheterization	93451	5827	1049 (616)	851 (652 - 1292)	1.98	0.59	8857 (6852)	7497 (4623 - 11607)	2.51	0.77
	Invasive coronary angiogram with left heart catheterization	93458	12047	1255 (724)	1044 (801 - 1444)	1.8	0.58	9257 (6719)	8066 (4798 - 12007)	2.5	0.73
Stress Testing	SPECT* imaging	78452	21284	537 (369)515	427 (333 - 599)	1.8	0.69	2087 (4242)	1336 (695 - 2442)	3.51	2.03
	Myocardial PET* stress test	78492	1247	1076 (684)	967 (649 - 1301)	2	0.64	3518 (2336)	3104 (1733 - 4387)	2.53	0.66
	Stress test supervision, interpretation, and report	93015	12098	134 (82)	115 (85 - 156)	1.83	0.61	1089 (7483)	117 (85 - 282)	3.32	6.87
	Exercise stress echocardiogram	93351	10245	306 (172)	253 (195 - 354)	1.82	0.56	1311 (4456)	639 (374 - 1191)	3.18	3.4

© 2026 Phillips AP et al. *JAMA Network Open*.

Note: This data represents April 2025 TiC data from ClarifyHealth, which aggregated price data for the 2023 contract year. Prices reflect the "allowed amount," which is the amount negotiated between an insurer and provider for a given Current Procedural Terminology (CPT) code (distinct from the "chargemaster rate"). See eTable 2 in the Supplement for this table disaggregated by commercial payer.

*Glossary of abbreviations:

CCTA = Coronary computed tomography angiography; ECG = Electrocardiogram; ICD = Implantable cardioverter-defibrillator; ILR = Implantable loop recorder; LAAO = Left atrial appendage occlusion; LVAD = Left ventricular assist device; MRI = Magnetic resonance imaging; PAP = Pulmonary artery pressure; PCI = Percutaneous coronary intervention; PET = Positron emission tomography; SPECT = Single photon emission computed tomography; TAVR = Transcatheter aortic valve replacement; TMVR = Transcatheter mitral valve replacement; VT = Ventricular tachycardia

© 2026 Phillips AP et al. *JAMA Network Open*.

eTable 2. Mean Prices and Price Variation for 32 Common Cardiology Services by Payer

				Professional Prices					Facility Prices				
**Description**	**Service Code**	**Payer Source**	**Unique Providers**	**Unique Price Points**	**Mean (SD)**	**Median (interquartile ratio)**	**Ratio of 75th to 25th %tiles**	**Coefficient of Variation**	**Unique Price Points**	**Mean (SD)**	**Median (interquartile ratio)**	**Ratio of 75th to 25th %tiles**	**Coefficient of Variation**
**Procedure Category: Cardiac Electrophysiology Evaluation**													
Electrocardiogram	93010	All Four Payers	29944	472783	16 (11)	13 (10 - 19)	1.98	0.66					
		Aetna	12610	42447	7 (5)	6 (5 - 8)	1.71	0.71					
		BlueCross BlueShield	26710	247323	18 (11)	14 (10 - 20)	2.02	0.64					
		Cigna	25105	66505	17 (11)	14 (11 - 20)	1.83	0.63					
		UnitedHealthcare	26811	116508	17 (11)	15 (11 - 20)	1.91	0.62					
30-day ECG monitor	93228	All Four Payers	11236	134721	46 (27)	39 (30 - 53)	1.77	0.58					
		Aetna	4867	14238	39 (19)	30 (25 - 46)	1.85	0.5					
		BlueCross BlueShield	9616	61995	47 (27)	38 (31 - 52)	1.7	0.59					
		Cigna	9706	19912	45 (21)	39 (32 - 54)	1.7	0.46					
		UnitedHealthcare	10621	38576	49 (30)	42 (32 - 57)	1.79	0.62					
Dual chamber pacemaker programming	93280	All Four Payers	10355	123008	82 (45)	69 (52 - 94)	1.81	0.55					
		Aetna	4265	11908	56 (26)	48 (39 - 67)	1.72	0.46					
		BlueCross BlueShield	8788	59123	87 (48)	74 (57 - 100)	1.77	0.55					
		Cigna	8849	17292	84 (46)	71 (56 - 96)	1.73	0.54					
		UnitedHealthcare	9788	34685	79 (41)	68 (50 - 93)	1.87	0.52					

© 2026 Phillips AP et al. *JAMA Network Open*.

Single chamber ICD programming	93282	All Four Payers	6313	73944	87 (46)	74 (56 - 101)	1.81	0.53						
		Aetna	2593	7249	73 (34)	62 (51 - 91)	1.8	0.46						
		BlueCross BlueShield	5375	35272	91 (49)	78 (60 - 105)	1.74	0.54						
		Cigna	5321	10463	89 (47)	74 (59 - 103)	1.74	0.53						
		UnitedHealthcare	5990	20960	83 (43)	73 (53 - 96)	1.83	0.51						
Remote interrogation - pacemaker	93294	All Four Payers	8726	102801	55 (30)	47 (36 - 62)	1.76	0.54						
		Aetna	3526	9893	45 (21)	38 (31 - 54)	1.72	0.46						
		BlueCross BlueShield	7487	48577	57 (35)	46 (35 - 64)	1.81	0.61						
		Cigna	7390	14687	56 (26)	49 (39 - 65)	1.65	0.47						
		UnitedHealthcare	8278	29644	55 (24)	51 (37 - 64)	1.7	0.43						
Remote interrogation - ICD	93295	All Four Payers	7702	90397	84 (56)	69 (52 - 97)	1.85	0.67						
		Aetna	3077	8581	67 (37)	58 (42 - 74)	1.76	0.55						
		BlueCross BlueShield	6614	42881	89 (65)	69 (53 - 97)	1.83	0.73						
		Cigna	6507	12909	87 (54)	75 (53 - 103)	1.94	0.62						
		UnitedHealthcare	7319	26026	81 (45)	73 (56 - 96)	1.7	0.55						
**Procedure: Cardiac Electrophysiology Procedures**														
Dual-chamber permanent pacemaker insertion	33208	All Four Payers	4967	63274	954 (662)	801 (601 - 1104)	1.84	0.69	6833	17178 (15573)	13035 (6920 - 22132)	3.2	0.91
		Aetna	2244	6415	554 (901)	344 (261 - 496)	1.9	1.62	1818	8130 (9426)	6366 (2775 - 10714)	3.86	1.16

© 2026 Phillips AP et al. *JAMA Network Open*.

		BlueCross BlueShield	4356	31071	980 (537)	812 (625 - 1142)	1.83	0.55	2447	18373 (14672)	14917 (8499 - 22951)	2.7	0.8
		Cigna	4270	8827	1027 (872)	815 (661 - 1099)	1.66	0.85	178	19672 (16619)	14989 (7857 - 28752)	3.66	0.84
		UnitedHealthcare	4701	16961	1020 (580)	885 (665 - 1179)	1.77	0.57	2390	22652 (17054)	17678 (11435 - 30304)	2.65	0.75
ICD placement	33249	All Four Payers	3670	45030	1626 (1378)	1359 (1000 - 1915)	1.91	0.85	6342	34456 (36080)	22544 (8150 - 49877)	6.12	1.05
		Aetna	1606	4569	961 (2065)	610 (460 - 935)	2.04	2.15	1660	9490 (10246)	6674 (3069 - 11402)	3.72	1.08
		BlueCross BlueShield	3216	21455	1575 (911)	1314 (1007 - 1850)	1.84	0.58	2316	41668 (41865)	32502 (10898 - 57758)	5.3	1
		Cigna	3167	6541	1827 (2017)	1448 (1175 - 1993)	1.7	1.1	172	32373 (27081)	28352 (11932 - 44019)	3.69	0.84
		UnitedHealthcare	3491	12465	1853 (1222)	1581 (1182 - 2111)	1.79	0.66	2194	45895 (33455)	36269 (18673 - 66880)	3.58	0.73
ILR placement	33285	All Four Payers	5625	66589	5819 (5068)	4348 (2853 - 7374)	2.58	0.87	7168	11487 (10038)	9291 (4685 - 15081)	3.22	0.87
		Aetna	2328	6604	1697 (967)	1319 (1153 - 1788)	1.55	0.57	1885	4707 (3812)	3953 (2058 - 6218)	3.02	0.81
		BlueCross BlueShield	4788	31346	5333 (5309)	3581 (2835 - 5412)	1.91	1	2604	13328 (11585)	11032 (6375 - 16173)	2.54	0.87
		Cigna	4741	9614	4150 (3642)	3458 (2675 - 4782)	1.79	0.88	118	10265 (6601)	9319 (6000 - 13378)	2.23	0.64

© 2026 Phillips AP et al. *JAMA Network Open*.

		UnitedHealthcare	5351	19025	8893 (4393)	7878 (6014 - 10387)	1.73	0.49	2561	14661 (9247)	12545 (8374 - 18424)	2.2	0.63
LAAO	33340	All Four Payers	2203	25744	1389 (766)	1201 (903 - 1666)	1.84	0.55	937	8072 (8113)	5922 (3021 - 9751)	3.23	1.01
		Aetna	908	2612	585 (265)	497 (394 - 685)	1.74	0.45	310	7954 (5293)	7420 (4634 - 11002)	2.37	0.67
		BlueCross BlueShield	1865	11969	1469 (818)	1191 (963 - 1709)	1.78	0.56	257	9992 (11317)	4611 (1916 - 14100)	7.36	1.13
		Cigna	1849	3632	1438 (676)	1258 (1020 - 1683)	1.65	0.47					
		UnitedHealthcare	2112	7531	1518 (667)	1366 (1065 - 1761)	1.65	0.44	363	6698 (6880)	4828 (3032 - 7323)	2.42	1.03
Cardioversion	92960	All Four Payers	14272	171445	283 (189)	241 (171 - 333)	1.95	0.67	7016	2369 (4556)	1650 (816 - 2698)	3.3	1.92
		Aetna	5798	16686	99 (52)	79 (64 - 112)	1.74	0.53	707	580 (2269)	118 (68 - 228)	3.38	3.91
		BlueCross BlueShield	12460	80043	290 (206)	230 (170 - 313)	1.84	0.71	2800	2423 (6743)	1192 (756 - 1986)	2.63	2.78
		Cigna	12308	25258	271 (155)	238 (183 - 305)	1.66	0.57	189	1607 (1996)	985 (503 - 1802)	3.58	1.24
		UnitedHealthcare	13523	49458	340 (165)	307 (232 - 396)	1.7	0.49	3320	2747 (1831)	2419 (1626 - 3431)	2.11	0.67
Supraventricular tachycardia ablation	93653	All Four Payers	3059	35646	1630 (1053)	1374 (1000 - 1929)	1.93	0.65	5410	23228 (42762)	16191 (8497 - 30234)	3.56	1.84
		Aetna	1191	3271	756 (926)	468 (377 - 772)	2.04	1.22	1477	9570 (8038)	7839 (3572 - 13836)	3.87	0.84
		BlueCross BlueShield	2734	17331	1710 (1151)	1387 (1037 - 2023)	1.95	0.67	1864	32852 (68315)	23549 (11737 - 45805)	3.9	2.08

© 2026 Phillips AP et al. *JAMA Network Open*.

		Cigna	2580	4964	1608 (764)	1396 (1121 - 1874)	1.67	0.47	223	26750 (19197)	21838 (15512 - 35800)	2.31	0.72
		UnitedHealthcare	2913	10080	1787 (893)	1558 (1203 - 2063)	1.71	0.5	1846	24013 (17527)	19022 (12163 - 31644)	2.6	0.73
VT ablation	93654	All Four Payers	1838	21603	2117 (1240)	1824 (1311 - 2579)	1.97	0.59	3207	24152 (52864)	16274 (8529 - 31216)	3.66	2.19
		Aetna	683	1883	763 (433)	579 (465 - 924)	1.99	0.57	877	9733 (8099)	7964 (3670 - 13950)	3.8	0.83
		BlueCross BlueShield	1619	10705	2218 (1308)	1880 (1344 - 2700)	2.01	0.59	1095	34986 (86676)	23881 (11745 - 47568)	4.05	2.48
		Cigna	1542	2960	2125 (1025)	1816 (1470 - 2531)	1.72	0.48	141	25891 (17583)	21702 (15651 - 34616)	2.21	0.68
		UnitedHealthcare	1748	6055	2357 (1117)	2064 (1608 - 2740)	1.7	0.47	1094	24641 (17330)	19258 (12037 - 32574)	2.71	0.7
Atrial fibrillation ablation	93656	All Four Payers	3106	36585	2101 (1348)	1780 (1298 - 2526)	1.95	0.64	5328	23462 (42946)	16372 (8800 - 30890)	3.51	1.83
		Aetna	1195	3319	871 (765)	568 (466 - 948)	2.03	0.88	1459	9681 (7995)	7964 (3633 - 13966)	3.84	0.83
		BlueCross BlueShield	2781	17920	2201 (1470)	1780 (1339 - 2619)	1.96	0.67	1822	33377 (69013)	23881 (12356 - 46362)	3.75	2.07
		Cigna	2636	5127	2081 (987)	1804 (1451 - 2478)	1.71	0.47	224	27259 (19092)	22214 (15623 - 36318)	2.32	0.7
		UnitedHealthcare	2953	10219	2336 (1212)	2025 (1592 - 2710)	1.7	0.52	1823	24114 (16850)	19083 (12252 - 32038)	2.62	0.7
**Procedure: Diagnostic Imaging**													

© 2026 Phillips AP et al. *JAMA Network Open*.

Cardiac MRI	75561	All Four Payers	3869	52316	686 (550)	526 (362 - 810)	2.24	0.8	2874	1614 (4335)	1037 (691 - 1719)	2.49	2.69
		Aetna	1784	5957	520 (362)	410 (280 - 633)	2.26	0.7	1256	1188 (793)	959 (589 - 1659)	2.82	0.67
		BlueCross BlueShield	3409	25879	782 (666)	563 (372 - 926)	2.49	0.85	1364	2098 (6203)	1139 (754 - 1877)	2.49	2.96
		Cigna	3229	7659	581 (390)	506 (342 - 729)	2.13	0.67	214	1141 (769)	908 (695 - 1355)	1.95	0.67
		UnitedHealthcare	3317	12821	631 (381)	521 (387 - 785)	2.03	0.6	40	1012 (319)	1010 (868 - 1087)	1.25	0.32
Coronary calcium scan	75571	All Four Payers	7971	133961	135 (121)	100 (71 - 157)	2.2	0.9	4493	863 (4834)	254 (149 - 572)	3.85	5.6
		Aetna	4022	15902	123 (131)	89 (67 - 142)	2.14	1.06	1899	613 (622)	326 (140 - 946)	6.73	1.01
		BlueCross BlueShield	7078	72019	144 (140)	98 (71 - 163)	2.3	0.97	2174	1140 (6895)	243 (150 - 432)	2.89	6.05
		Cigna	6649	20299	117 (72)	103 (67 - 147)	2.2	0.61	341	643 (1302)	226 (166 - 414)	2.5	2.02
		UnitedHealthcare	5869	25741	131 (77)	110 (78 - 156)	1.99	0.59	79	214 (98)	206 (144 - 249)	1.73	0.46
CCTA	75574	All Four Payers	9042	134263	503 (408)	388 (273 - 568)	2.08	0.81	4998	1153 (3302)	686 (456 - 1120)	2.46	2.86
		Aetna	4274	14997	349 (202)	276 (232 - 410)	1.77	0.58	2168	862 (606)	672 (402 - 1190)	2.96	0.7
		BlueCross BlueShield	7818	68629	554 (497)	388 (283 - 605)	2.14	0.9	2348	1439 (4724)	693 (490 - 1069)	2.18	3.28
		Cigna	7693	20621	464 (294)	415 (272 - 579)	2.13	0.63	386	1221 (1495)	725 (524 - 1097)	2.09	1.22
		UnitedHealthcare	7398	30016	489 (284)	419 (292 - 581)	1.99	0.58	96	447 (195)	427 (333 - 507)	1.52	0.44
Transthoracic echocardiogram	93306	All Four Payers	30767	433579	287 (175)	231 (173 - 341)	1.97	0.61	6644	1325 (5126)	643 (318 - 1229)	3.87	3.87
		Aetna	13744	43326	235 (152)	170 (139 - 259)	1.87	0.65	2002	498 (668)	280 (183 - 486)	2.66	1.34
		BlueCross BlueShield	27133	219721	302 (191)	232 (179 - 364)	2.04	0.63	4200	1740 (6386)	841 (478 - 1494)	3.13	3.67
		Cigna	26041	60681	274 (156)	233 (180 - 325)	1.8	0.57	261	1025 (960)	696 (446 - 1169)	2.62	0.94
		UnitedHealthcare	27834	109851	282 (154)	243 (180 - 333)	1.85	0.55	181	1280 (734)	1227 (818 - 1509)	1.84	0.57

© 2026 Phillips AP et al. *JAMA Network Open*.

Transesophageal echocardiogram	93312	All Four Payers	14183	174003	322 (194)	272 (206 - 374)	1.82	0.6	4032	1251 (4900)	642 (365 - 1193)	3.27	3.92
		Aetna	5930	17361	183 (105)	144 (118 - 206)	1.75	0.57	1138	466 (718)	278 (151 - 476)	3.16	1.54
		BlueCross BlueShield	12315	81506	345 (209)	274 (220 - 385)	1.76	0.61	2634	1589 (5994)	845 (494 - 1381)	2.79	3.77
		Cigna	12331	25777	322 (165)	282 (218 - 374)	1.72	0.51	173	1215 (1787)	751 (505 - 1169)	2.31	1.47
		UnitedHealthcare	13388	49359	334 (185)	292 (225 - 387)	1.72	0.55	87	1387 (577)	1284 (1059 - 1527)	1.44	0.42
Carotid ultrasound	93880	All Four Payers	26378	475712	227 (163)	180 (137 - 253)	1.85	0.72	7377	830 (4409)	419 (222 - 692)	3.12	5.31
		Aetna	12161	47905	148 (90)	117 (103 - 152)	1.47	0.61	1716	333 (338)	225 (127 - 382)	2.99	1.01
		BlueCross BlueShield	23220	252143	235 (191)	179 (141 - 241)	1.71	0.81	3860	1143 (5954)	507 (333 - 796)	2.39	5.21
		Cigna	22859	75913	235 (130)	200 (147 - 285)	1.94	0.55	1150	841 (2161)	529 (371 - 752)	2.03	2.57
		UnitedHealthcare	21257	99751	238 (119)	210 (154 - 287)	1.86	0.5	651	261 (168)	190 (190 - 190)	1	0.64
**Procedure: Interventional Cardiology Procedures**													
PAP sensor placement	33289	All Four Payers	517	6140	621 (333)	524 (417 - 728)	1.75	0.54	1156	24973 (32560)	14993 (8109 - 33232)	4.1	1.3
		Aetna	208	617	288 (117)	249 (188 - 363)	1.93	0.41	356	7576 (4794)	7668 (4462 - 10205)	2.29	0.63
		BlueCross BlueShield	462	2782	643 (356)	515 (424 - 724)	1.71	0.55	345	34091 (48334)	22585 (10107 - 47346)	4.68	1.42
		Cigna	436	894	657 (301)	591 (454 - 791)	1.74	0.46					
		UnitedHealthcare	499	1847	681 (295)	606 (496 - 804)	1.62	0.43	437	31889 (22653)	25636 (15759 - 39443)	2.5	0.71
TAVR	33361	All Four Payers	2098	25792	2177 (1244)	1886 (1336 - 2642)	1.98	0.57	150	7454 (6672)	6018 (3467 - 8975)	2.59	0.9

© 2026 Phillips AP et al. *JAMA Network Open*.

		Aetna	877	2486	886 (409)	747 (567 - 1057)	1.86	0.46					
		BlueCross BlueShield	1804	12608	2205 (1269)	1816 (1336 - 2642)	1.98	0.58					
		Cigna	1776	3469	2360 (1170)	2052 (1619 - 2807)	1.73	0.5					
		UnitedHealthcare	1983	7229	2484 (1144)	2239 (1704 - 2885)	1.69	0.46					
TMVR	33418	All Four Payers	794	10003	3379 (1913)	2817 (1996 - 4168)	2.09	0.57	81	6191 (7348)	4704 (2606 - 6524)	2.5	1.19
		Aetna	326	960	1421 (703)	1173 (884 - 1767)	2	0.49					
		BlueCross BlueShield	670	4913	3525 (1997)	2729 (2012 - 4483)	2.23	0.57					
		Cigna	697	1406	3409 (1591)	3004 (2455 - 3968)	1.62	0.47					
		UnitedHealthcare	756	2724	3790 (1794)	3361 (2549 - 4454)	1.75	0.47					
Intra-aortic balloon pump placement	33967	All Four Payers	3497	44691	501 (575)	395 (295 - 547)	1.86	1.15	469	13785 (14984)	8086 (3963 - 18098)	4.57	1.09
		Aetna	1664	4904	472 (1464)	159 (123 - 236)	1.92	3.1	137	5124 (4694)	4951 (651 - 7146)	10.98	0.92
		BlueCross BlueShield	3034	21101	484 (260)	393 (312 - 555)	1.78	0.54	137	7005 (8334)	4868 (893 - 8093)	9.07	1.19
		Cigna	3012	6224	490 (265)	418 (328 - 559)	1.7	0.54					
		UnitedHealthcare	3317	12462	549 (435)	449 (340 - 599)	1.76	0.79	192	24999 (16410)	21320 (12484 - 31862)	2.55	0.66
Percutaneous LVAD	33990	All Four Payers	2614	31904	665 (368)	576 (425 - 803)	1.89	0.55	846	6754 (7267)	4799 (2397 - 8630)	3.6	1.08

© 2026 Phillips AP et al. *JAMA Network Open*.

placement to left heart		Aetna	1126	3213	270 (137)	218 (177 - 297)	1.68	0.51	245	6239 (6735)	4705 (3071 - 7869)	2.56	1.08
		BlueCross BlueShield	2224	14972	653 (358)	544 (416 - 769)	1.85	0.55	251	7908 (8369)	6100 (1646 - 11580)	7.03	1.06
		Cigna	2227	4670	718 (338)	645 (504 - 846)	1.68	0.47					
		UnitedHealthcare	2494	9049	797 (356)	727 (561 - 921)	1.64	0.45	338	5910 (5664)	4584 (2598 - 7508)	2.89	0.96
PCI with stent placement	92928	All Four Payers	9079	114141	1034 (718)	882 (655 - 1194)	1.82	0.69	4054	16080 (12945)	12710 (7266 - 20385)	2.81	0.81
		Aetna	3927	11378	571 (1220)	291 (252 - 438)	1.74	2.14	1041	8101 (6784)	7159 (2914 - 11412)	3.92	0.84
		BlueCross BlueShield	7950	53316	1065 (611)	885 (683 - 1218)	1.78	0.57	1376	20966 (15342)	16367 (10690 - 28930)	2.71	0.73
		Cigna	7844	16563	1053 (626)	904 (741 - 1211)	1.63	0.59	326	19968 (12134)	18012 (10059 - 24861)	2.47	0.61
		UnitedHealthcare	8612	32884	1132 (620)	979 (749 - 1307)	1.74	0.55	1311	16320 (10901)	13566 (8140 - 20309)	2.49	0.67
Right heart catheterization	93451	All Four Payers	5827	70512	1049 (616)	851 (652 - 1292)	1.98	0.59	5526	8857 (6852)	7497 (4623 - 11607)	2.51	0.77
		Aetna	2487	6824	934 (438)	743 (627 - 1166)	1.86	0.47	1478	6188 (4837)	5574 (2915 - 8269)	2.84	0.78
		BlueCross BlueShield	5146	33456	1034 (677)	814 (588 - 1333)	2.27	0.66	1876	9007 (8305)	7129 (4277 - 11632)	2.72	0.92
		Cigna	4891	10019	1060 (562)	868 (709 - 1216)	1.71	0.53	323	12178 (7558)	10875 (7112 - 14402)	2.02	0.62

© 2026 Phillips AP et al. *JAMA Network Open*.

		UnitedHealthcare	5497	20213	1106 (580)	952 (714 - 1331)	1.86	0.52	1849	10258 (5669)	9219 (6400 - 12807)	2	0.55
Invasive coronary angiogram with left heart catheterization	93458	All Four Payers	12047	154119	1255 (724)	1044 (801 - 1444)	1.8	0.58	10150	9257 (6719)	8066 (4798 - 12007)	2.5	0.73
		Aetna	5281	15398	1108 (640)	870 (762 - 1350)	1.77	0.58	2640	7645 (5680)	6979 (3582 - 10068)	2.81	0.74
		BlueCross BlueShield	10561	73225	1260 (795)	1010 (757 - 1428)	1.89	0.63	3546	9306 (7640)	7708 (4334 - 11988)	2.77	0.82
		Cigna	10464	22612	1254 (612)	1070 (883 - 1442)	1.63	0.49	552	12047 (7561)	10718 (6773 - 14398)	2.13	0.63
		UnitedHealthcare	11352	42884	1298 (672)	1153 (858 - 1512)	1.76	0.52	3412	10003 (5972)	8997 (6167 - 12447)	2.02	0.6
SPECT imaging	78452	All Four Payers	21284	295544	537 (369)	427 (333 - 599)	1.8	0.69	7371	2087 (4242)	1336 (695 - 2442)	3.51	2.03
		Aetna	10596	33963	439 (234)	349 (290 - 517)	1.78	0.53	2377	910 (734)	648 (410 - 1086)	2.65	0.81
		BlueCross BlueShield	18639	144546	573 (437)	441 (358 - 595)	1.66	0.76	3961	2640 (5488)	1721 (1121 - 2868)	2.56	2.08
		Cigna	18227	43025	488 (279)	427 (309 - 599)	1.94	0.57	902	2611 (2768)	1583 (1004 - 3644)	3.63	1.06
		UnitedHealthcare	18907	74010	540 (303)	442 (338 - 634)	1.88	0.56	131	3138 (1517)	3220 (2218 - 3885)	1.75	0.48
**Procedure: Stress Testing**													
Myocardial PET stress test	78492	All Four Payers	1247	15713	1076 (684)	967 (649 - 1301)	2	0.64	155	3518 (2336)	3104 (1733 - 4387)	2.53	0.66
		Aetna	719	2127	658 (487)	617 (290 - 774)	2.67	0.74	87	3275 (2534)	2957 (1343 - 4467)	3.33	0.77

© 2026 Phillips AP et al. *JAMA Network Open*.

		BlueCross BlueShield	1071	7106	1261 (633)	1137 (817 - 1538)	1.88	0.5	59	4039 (2003)	3717 (2853 - 4266)	1.5	0.5
		Cigna	1149	2413	579 (499)	444 (253 - 764)	3.02	0.86					
		UnitedHealthcare	1150	4067	1265 (709)	1050 (821 - 1567)	1.91	0.56					
Stress test supervision, interpretation, and report	93015	All Four Payers	12098	150290	134 (82)	115 (85 - 156)	1.83	0.61	149	1089 (7483)	117 (85 - 282)	3.32	6.87
		Aetna	5735	16798	66 (39)	53 (39 - 76)	1.94	0.59	52	158 (158)	82 (60 - 178)	2.95	1
		BlueCross BlueShield	10239	70256	148 (87)	120 (95 - 164)	1.72	0.59	93	1647 (9446)	167 (114 - 318)	2.79	5.74
		Cigna	10539	21344	139 (84)	117 (90 - 156)	1.73	0.6					
		UnitedHealthcare	11440	41892	137 (70)	121 (91 - 162)	1.78	0.51					
Exercise stress echocardiogram	93351	All Four Payers	10245	127087	306 (172)	253 (195 - 354)	1.82	0.56	1797	1311 (4456)	639 (374 - 1191)	3.18	3.4
		Aetna	4319	12511	258 (131)	198 (168 - 309)	1.84	0.51	510	492 (725)	287 (202 - 486)	2.41	1.48
		BlueCross BlueShield	9187	63540	314 (189)	250 (200 - 345)	1.72	0.6	1160	1700 (5482)	827 (496 - 1332)	2.69	3.23
		Cigna	8556	16749	309 (166)	257 (205 - 361)	1.77	0.54	68	816 (473)	669 (545 - 976)	1.79	0.58
		UnitedHealthcare	9569	34287	308 (152)	268 (204 - 359)	1.76	0.5	59	1336 (389)	1432 (1083 - 1522)	1.4	0.29

Note: This data represents April 2025 TiC data from ClarifyHealth, which aggregates price data for the 2023 contract year. Prices reflect the "allowed amount," which is the amount negotiated between an insurer and provider for a given Current Procedural Terminology (CPT) code (distinct from the "chargemaster rate").

© 2026 Phillips AP et al. *JAMA Network Open*.
