# Supplementary material for: Commercial Price Variation for Common Cardiovascular Services Across 4 Major US Insurers
Source: JAMA Netw Open. 2026 Jul 16;9(7):e2623326. doi: 10.1001/jamanetworkopen.2026.23326 (PMC13377394; doi:10.1001/jamanetworkopen.2026.23326)
Supplement: Supplement 2. — Data Sharing Statement [file jamanetwopen-e2623326-s002.pdf]

Data Sharing Statement

Philips. Commercial Price Variation for Common Cardiovascular Services Across 4 Major US Insurers. *JAMA Netw Open*. Published July 16, 2026.
doi:10.1001/jamanetworkopen.2026.23326

Data
Data available: No
